# Serpentine Thrombus in Right Atrium: A Tell-Tale Sign of Venous Thromboembolism

**DOI:** 10.7759/cureus.20336

**Published:** 2021-12-10

**Authors:** Vidyashree Hulkoti, Sandeep Kamat, Sourya Acharya, Samarth Shukla, Sunil Kumar, Dhruv Talwar

**Affiliations:** 1 Department of Medicine, Jawaharlal Nehru Medical College, Datta Meghe Institute of Medical Sciences (Deemed to be university), Wardha, IND; 2 Department of Cardiology, Topiwala National Medical College & BYL Nair Charitable Hospital, Mumbai, IND; 3 Department of Pathology, Jawaharlal Nehru Medical College, Datta Meghe Institute of Medical Sciences (Deemed to be university), Wardha, IND

**Keywords:** heparin, virchow s triad, pulmonary embolism, deep vein thrombosis, serpentine clot

## Abstract

Pulmonary embolism is linked with a remarkable rate of mortality, particularly when it is associated with hemodynamic instability, right atrial thrombus and related right ventricular dysfunction. In patients affected with pulmonary embolism, thrombolysis has been documented to be life-saving. The administration of thrombolytic agents ensures early resolution of the thrombus and prevents arrhythmia and cardiogenic shock. Streptokinase, urokinase and alteplase are among the three thrombolytic agents approved for the treatment of pulmonary embolism. In emergency situations, thrombectomy plays a crucial role.

## Introduction

Circulation of blood in a hemodynamically stable state ensures ideal equilibrium in the human body. Stasis of blood, vessel wall injury and hypercoagulability result in the formation of thrombus. This thrombus formed in the lower limb is known as deep vein thrombosis (DVT). Patients with DVT present with severe pain and swelling in the affected limb. In case of severe trauma or injury or increased activity, dislodgement of this thrombus causes free movement of the thrombus in the circulation [1]. This thrombus can get obstructed in any major vessel and can pathological conditions. Dislodgement of the thrombus in the right atrium (RA) results in pulmonary embolism [2]. Serpentine thrombus seen in the RA is very pathognomonic of thrombus being dislodged from the deep veins of the lower limbs. Pulmonary embolism can be life-threatening as it can cause severe arrhythmias, cardiogenic shock and death. Hence, prompt treatment is required. Hereby, we discuss the various management protocol for pulmonary embolism patients.

## Case presentation

A 32-year-old young male, a teacher by profession, presented with complaints of sudden onset of severe chest pain and breathlessness for five days. The patient also had complaints of left lower limb swelling for 15 days.

Breathlessness was sudden in onset, initially, the patient experienced slight breathlessness after walking 500m, which progressed to breathlessness at rest in a span of three days. The patient had a dry cough associated with breathlessness. No history of orthopnoea, paroxysmal nocturnal dyspnea (PND) or palpitation was present. The patient also complained of left lower limb swelling for 15 days.

**Figure 1:**
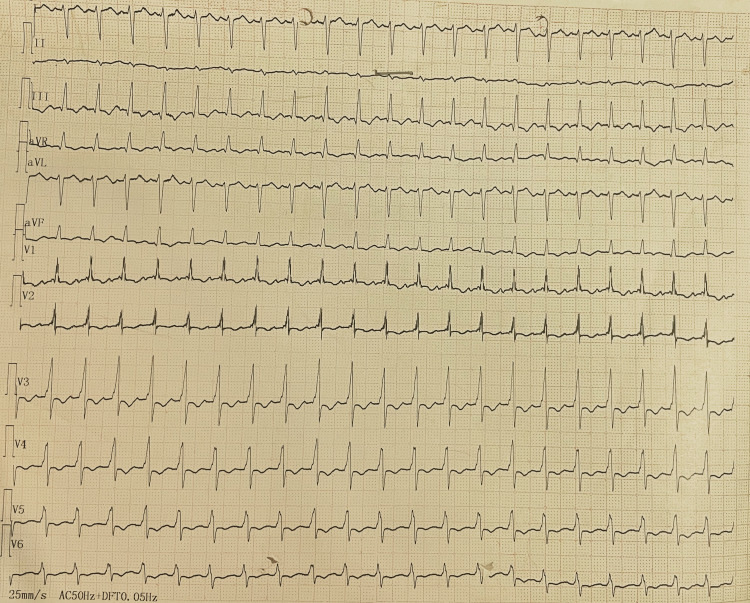
Sinus tachycardia (rate 122/min) and S1Q3T3 pattern

**Figure 2:**
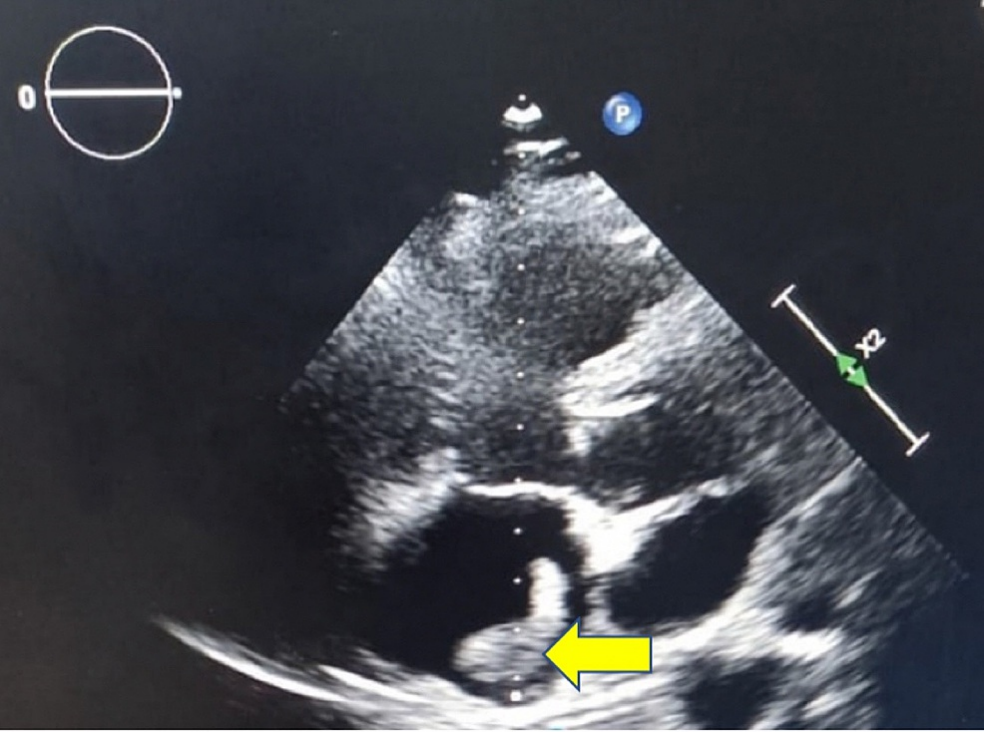
2D-echo suggestive of RA, RV dilatation with floating mass in RA approximately 1-2cm with severe pulmonary artery hypertension. RA - right atrium, RV - right ventricle

**Figure 3:**
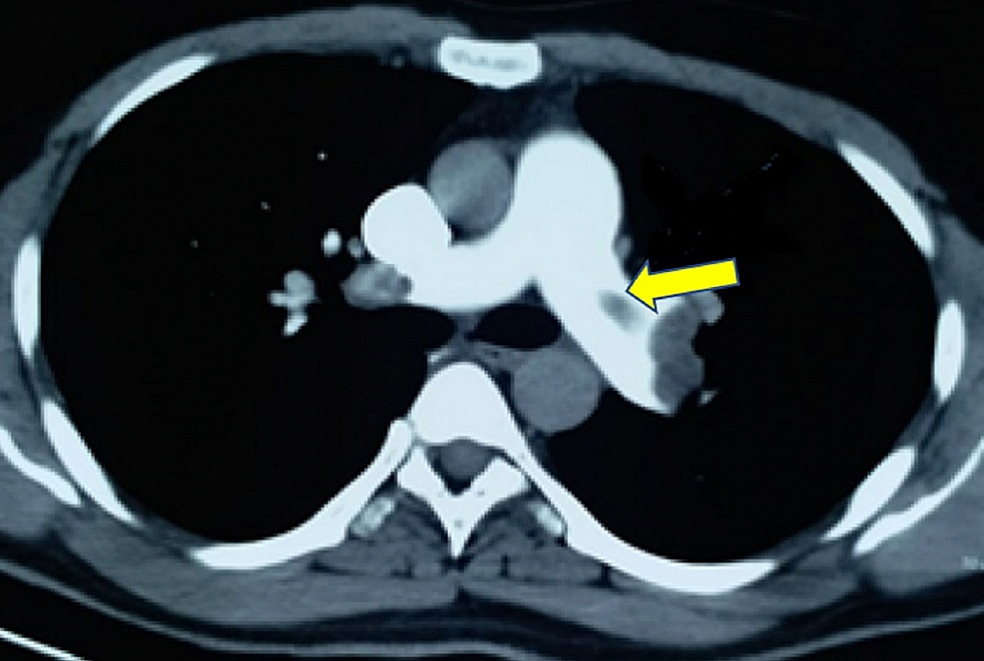
CT pulmonary angiogram showing thrombus in pulmonary artery

On examination, the patient was cyanosed with an increased respiratory rate of 28 breaths/min, SpO_2_ was 90%. heart rate - 122/min and blood pressure of 90/60mmHg. He was given high flow oxygen, intravenous fluid and dopamine infusion for hypotension. Electrocardiogram (ECG) done showed sinus tachycardia (rate 122/min) and S1Q3T3 pattern (Figure 1). Echocardiogram was done which showed dilated RA and right ventricle (RV) (Figure 2), floating thrombus in RA and impaired RV systolic function. CT pulmonary angiogram of the patient was done that showed the clear dimension of the clot present in the pulmonary artery (Figure 3).

All routine investigation - complete blood count, liver function test, kidney function test was done along with erythrocyte sedimentation rate, D-dimer, serum antinuclear antibody (ANA) and antineutrophil cytoplasmic antibody (ANCA), and serum homocysteine. Reports were suggestive of raised serum homocysteine level and positive D-dimer.

Doppler of left lower limb was done suggestive of left lower limb deep venous thrombosis.

In view of pulmonary embolism, injection heparin 5,000 IU units IV bolus followed by continuous infusion of 1,300 units/hr as started for 12 hours. As the patient improved infusion was stopped after 24 hours.

After 24 hours, cardiothoracic surgeon was consulted, and the patient was planned for embolectomy. On the establishment of total cardiopulmonary bypass, the pulmonary trunk was opened and the emboli were removed from the arteries. Post stabilization of thrombus, inferior vena cava (IVC) filter was placed and the risk of future thrombus was eliminated.

The patient had improved significantly during the hospital stay and was discharged comfortably.

## Discussion

DVT is the third most common cause of death after myocardial infarction and stroke. It accounts for 80 cases of DVT in 100,000 people. Among these DVT cases, a few develop pulmonary embolism [3].

As the deep veins of the lower limb are thick and slender, when the thrombus gets dislodged from these lower limbs this thrombus in the RA takes a serpentine shape. Hence, the serpentine thrombus seen in the RA is a tell-tale sign of the thrombus having origin from the deep veins. These serpentine thrombi account for 28% of death in hospitals caused by serpentine thrombus leading to pulmonary embolism.

Varied presentations of pulmonary embolism have been reported before [4,5]. Here, we have reported a case of a patient having serpentine pulmonary embolism due to venous thromboembolism and its management.

The Virchows triad plays a crucial role in the development of DVT. The endothelial damage, hypercoagulability and stasis of blood result in the formation of a thrombus that gets lodged in the veins of the lower limb resulting in pulmonary embolism. The various etiology responsible for this hypercoagulability is protein C, S deficiency, factor V Leiden mutation, raised serum homocysteine levels or it could be acquired due to cancer, pregnancy, chemotherapy, obesity [3]. This thrombus can get dislodged by trauma or activity and by circulation the thrombus can get clogged into the major arteries or can even reach the heart.

The thrombus from the deep veins of the lower limb results in a serpentine thrombus in the RA due to the retained shape of the thrombus.

**Figure 4:**
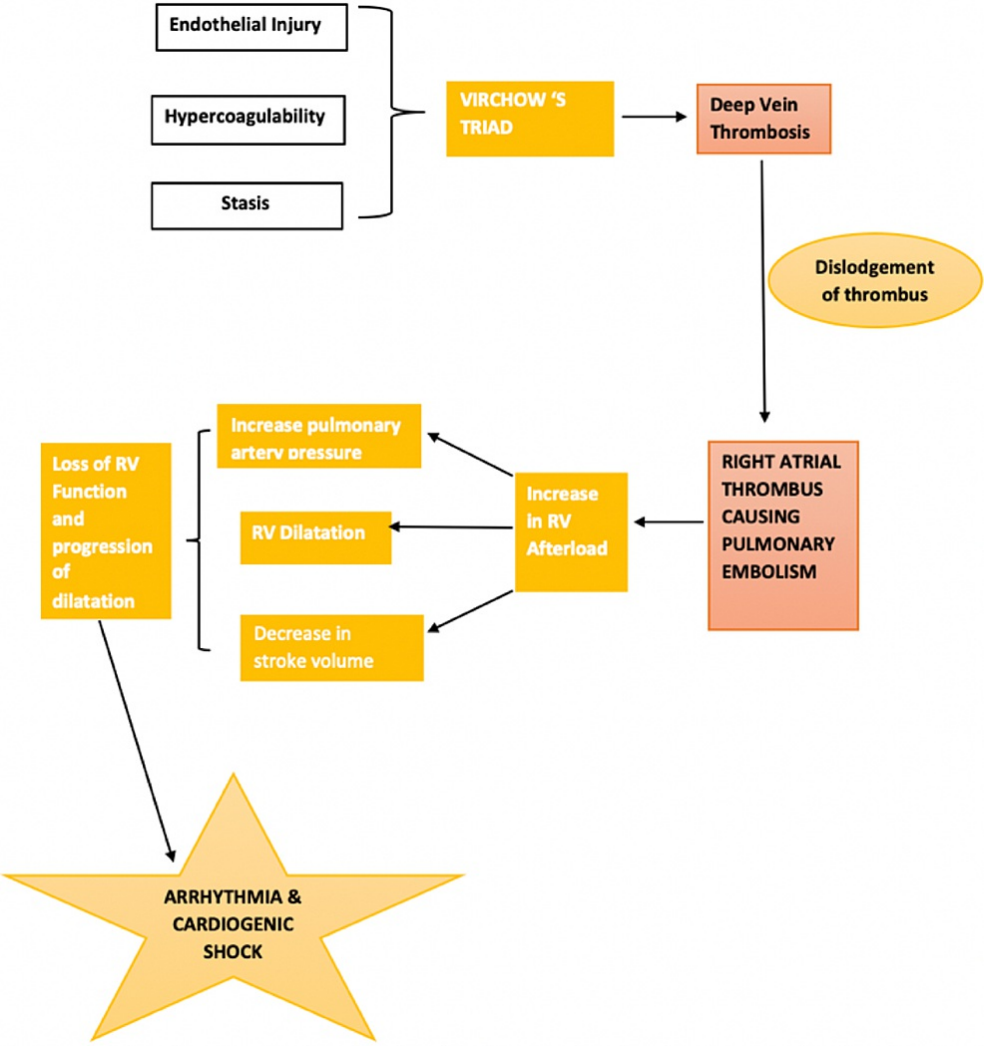
Pathophysiology of venous pulmonary embolism

The serpentine clots seen in the RA are the pathognomic signs of DVT [6]. This clot results in pulmonary embolism and commands urgent treatment. Acute pulmonary embolism causing this pulmonary vascular obstruction and vasoconstriction results in an increase in right ventricular afterload, thereby leading to a rise in pulmonary artery pressure, right ventricular dilatation and a decrease in the stroke volume. These myocardial changes lead to neurohumoral stimulation, increased oxygen demand and a decrease in right ventricular perfusion and thereby causing arrhythmias and hypotensive shock. Hence, prompt management of pulmonary embolism is crucial [7]. The pathophysiology of venous pulmonary embolism is shown in Figure 4.

Pulmonary embolism can be treated by thrombolysis or in emergency patients, thrombolectomy should be planned.

Three thrombolytics have been approved for the treatment of pulmonary embolism - streptokinase, urokinase and alteplase. These thrombolytic agents actively promote the hydrolysis of fibrin molecules, resulting in resolution of the thromboembolic occlusion leading to a reduction in the pulmonary artery pressure and resistance preventing the risk of arrhythmias and shock. There have been reports of catheter-directed thrombolysis successfully treating acute pulmonary embolism [8].

 A study by Rose et al. showed that in patients with PE with right heart thrombo-embolism, the mortality rate associated with anticoagulation therapy was 28.6%, surgical embolectomy was 23.8% and with thrombolysis was 11.3%. Hence, in patients with right atrial thrombus, thrombolysis demonstrated a better survival rate compared with surgery or anticoagulation therapy [9].

In case of emergency, surgical embolectomy is decisive for the management of pulmonary embolism patients. It is a confirmatory treatment for pulmonary embolism. However, operator expertise is recommended to ensure ideal results [10].

## Conclusions

Venous thromboembolism collectively includes DVT and pulmonary embolism. This venous thromboembolism constitutes the major global burden of disease. Pulmonary embolism although lethal, prompt diagnosis and management of this condition, ensures better livelihood and good prognosis with reduced mortality and morbidity.
